# Supplementary material for: Periprosthetic Joint Infection (PJI)—Results of One-Stage Revision with Antibiotic-Impregnated Cancellous Allograft Bone—A Retrospective Cohort Study
Source: Antibiotics (Basel). 2022 Feb 25;11(3):310. doi: 10.3390/antibiotics11030310 (PMC8944641; doi:10.3390/antibiotics11030310)
Supplement: Supplementary file 1 [file antibiotics-11-00310-s001.zip › antibiotics-1613727-supplementary.pdf]

antibiotics

MDPI

Supplementary Materials

Figure S1. Venn diagram (n = 124) demonstrating the overlap of patients diagnosed according to MSIS, EBJIS 2018, IDSA (including the clinician's judgement), IDSA (excluding the clinician's judgement), ICM 2013 and ICM 2018 (≙ the 2018 Definition of Periprosthetic Hip and Knee Infection).

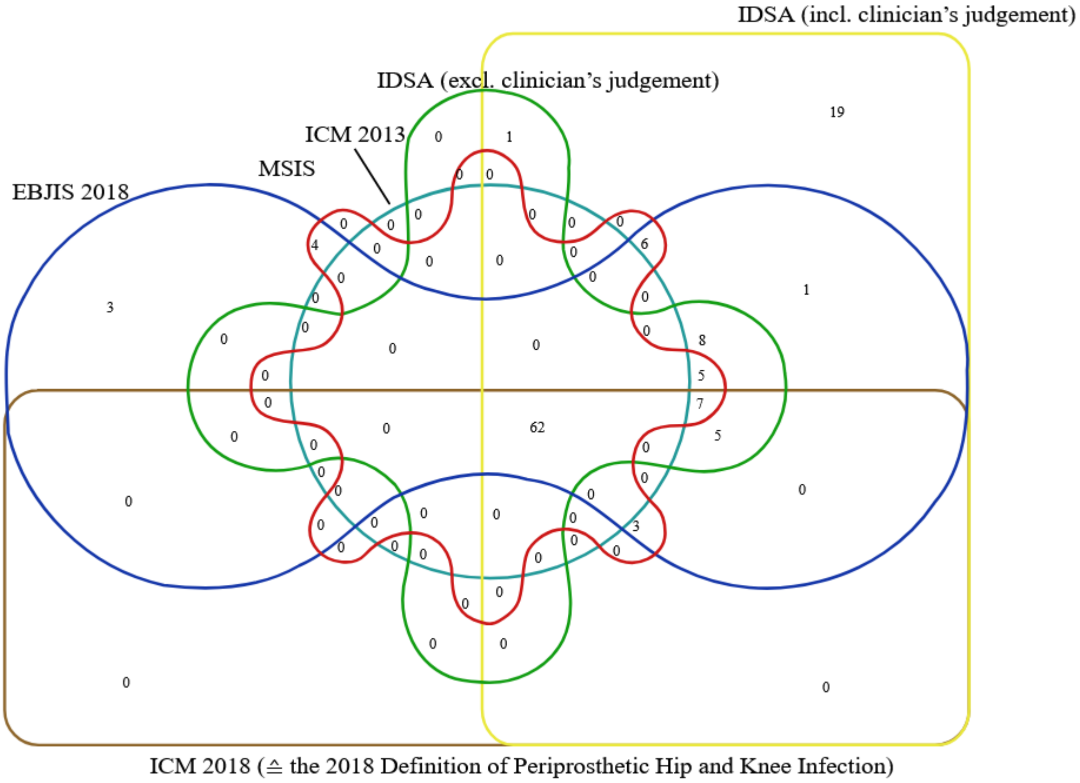

Figure S2. Patient questionnaire sent to international patients.

Patient Questionnaire

Please mark X according to your selection. If you have any questions about answering the questionnaire, please contact me or mark the answer "I don't know". Phone: XXXX Email: gregor.dersch@gmail.com

**1.1) On the XX.XX.XXXX you have had a revision surgery. Since then, did you have another surgery at your right/left knee/hip joint?**	☐ Yes<br>☐ No<br>☐ I don't know
**1.2) <u>If yes,</u> when was that?**<br>*(Multiple answers possible)*	DD | MM | YYYY

	DD | MM | YYYY<br>DD | MM | YYYY
**1.3) <u>If yes,</u> what has been done during that surgery (or those surgeries)?**<br>*(Multiple answers possible)*	☐ Lavage<br>☐ Synovectomy<br>☐ Total prosthesis replacement<br>☐ Partial prosthesis replacement<br>☐ Inlay replacement<br>☐ Patella implantation/-replacement<br>☐ Prosthesis removal<br>☐ Spacer implantation<br>☐ Joint stiffening (Arthrodesis)<br>☐ Amputation<br>☐ Fistula/ Sinus tract removal<br>☐ Negative pressure wound therapy/ Vacuum assisted closure (VAC)<br>☐ Open reduction of a joint dislocation<br>☐ Closed reduction of a joint dislocation<br>☐ Other ______________________<br>☐ I don't know
**2.1) Did you have a joint infection at your right/left knee/hip joint since XX.XX.XXXX?**	☐ Yes<br>☐ No<br>☐ I don't know
**2.2) <u>If yes,</u> when has it been diagnosed?**<br>*(Year and month are sufficient)*	DD | MM | YYYY
**2.3) <u>If yes</u>, how has it been diagnosed?**<br>*(Multiple answers possible)*	☐ Joint aspiration<br>☐ Intraoperative sample/microbial culture<br>☐ Blood analysis<br>☐ Symptoms<br>☐ Wound healing disorder<br>☐ Fistula/ Sinus tract<br>☐ Scintigraphy<br>☐ Radiology (X-Ray, MRI, CT,...)<br>☐ Other ______________________<br>☐ I don't know
**3.1) Has the patient passed away?**	☐ Yes

	☐ No
**3.2) <u>If yes,</u> when?**	DD | MM | YYYY
**4.1) Is there anything else that you want to tell us?**<br>*(Optional)*	

Figure S3. Patient questionnaire sent to national patients.

Patienten-Fragebogen

Zutreffendes bitte mit einem X ankreuzen. Sollte es Probleme beim Beantworten einer Frage geben, dann kontaktieren Sie mich bitte unter XXXX / gregor.dersch@gmail.com oder kreuzen Sie als Antwort "weiß ich nicht" an.

**1.1) Bei Ihnen wurde am XX.XX.XXXX am rechten/linken Kniegelenk/Hüftgelenk ein Prothesenwechsel durchgeführt. Wurden Sie seitdem nochmals an dieser Stelle operiert?**	☐ ja<br>☐ nein<br>☐ weiß ich nicht
**1.2) <u>Wenn ja,</u> wann war das?**<br>*(Mehrere Nennungen möglich)*	TT | MM | JJJJ<br>TT | MM | JJJJ<br>TT | MM | JJJJ
**1.3) <u>Wenn ja,</u> was wurde bei dieser/diesen Reoperation(en) durchgeführt?**<br>*(Gegebenenfalls mehreres ankreuzen)*	☐ Spülung<br>☐ Synovektomie<br>☐ Vollprothesenwechsel<br>☐ Teilprothesenwechsel<br>☐ Inlaywechsel<br>☐ Patellaimplantation/-wechsel<br>☐ Prothesenentfernung<br>☐ Spacerimplantation<br>☐ Gelenksversteifung (Athrodese)<br>☐ Amputation<br>☐ Fistelexzision (Fistelentfernung)<br>☐ Anlegen eines VAC-Verbandes<br>☐ Repositionierung mit Hautschnitt<br>☐ Repositionierung ohne Hautschnitt<br>☐ anderes ___________________

	☐ weiß ich nicht
**2.1) Hatten Sie seit dem XX.XX.XXXX eine Gelenksinfektion am rechten/linken Kniegelenk/Hüftgelenk?**	☐ ja<br>☐ nein<br>☐ weiß ich nicht
**2.2) Wenn ja, wann wurde diese festgestellt?**<br>*(Angabe des Jahres und des Monats reicht aus)*	TT | MM | JJJJ
**2.3) Wenn ja, wie wurde diese festgestellt?**<br>*(Gegebenenfalls mehreres ankreuzen)*	☐ Gelenkspunktion<br>☐ intraoperativer Abstrich<br>☐ Blutabnahme/Labor<br>☐ klinisch/Beschwerden/Symptome<br>☐ Wundheilungsstörung<br>☐ Fistel<br>☐ Szintigraphie<br>☐ radiologisches Verfahren(Röntgen, MRT, CT,...)<br>☐ anderes ___________________<br>☐ weiß ich nicht
**3.1) Ist der/die Patient/-in verstorben?**	☐ ja<br>☐ nein
**3.2) Wenn ja, wann?**	TT | MM | JJJJ
**4.1) Gibt es sonst noch etwas, das Sie uns mitteilen möchten?**<br>*(Optional)*	

Table S1. Identified microbial pathogens in 70 index revisions and 6 reinfections.

**Microorganisms**	**Index Revisions (n = 70)**	**Reinfections (n = 6)**
**Monomicrobial infections**	**32 (45.7%)**	**3 (50%)**
Candida albicans	1 (1.4%)	
Candida glabrata		1 (16.7%)
Propionibacterium acnes	2 (2.9%)	
Propionibacterium avidum	1 (1.4%)	
Staphylococcus aureus	1 (1.4%)	1 (16.7%)
Staphylococcus epidermidis	16 (22.9%)	
Staphylococcus capitis	1 (1.4%)	
Staphylococcus lugdunensis	1 (1.4%)	
Coagulase-negative *Staphylococcus* sp., unspecified	2 (2.9%)	
Group F *Streptococcus* sp.	1 (1.4%)	

Streptococcus parasanguis	1 (1.4%)	
Streptococcus sp., unspecified	1 (1.4%)	
Enterococcus faecium	1 (1.4%)	1 (16.7%)
Escherichia coli	1 (1.4%)	
Pseudomonas aeruginosa	2 (2.9%)	
**Polymicrobial infections**	**38 (54.3%)**	**2 (33.3%)**
Propionibacterium acnes + *S. epidermidis*	2 (2.9%)	
Propionibacterium sp. + *S. epidermidis*	2 (2.9%)	
Propionibacterium acnes + *S. capitis*	1 (1.4%)	
Propionibacterium sp. + *S. hominis* + *S. epidermidis*	1 (1.4%)	
S. caprae + *S. epidermidis*	1 (1.4%)	
S. warneri + *S. epidermidis*	1 (1.4%)	
S. epidermidis + mixed growth of CoNS	1 (1.4%)	
S. aureus + *S. epidermidis*	2 (2.9%)	
S. aureus + *S. hominis* + *S. epidermidis*	1 (1.4%)	
S. aureus + Viridans group *Streptococcus* sp. + *S. epidermidis*	1 (1.4%)	
Streptococcus agalactiae + *S. capitis*	1 (1.4%)	
Streptococcus thermophilus + Viridans group *Streptococcus* sp. + mixed growth of CoNS	1 (1.4%)	
Enterococcus faecalis + *S. aureus* + *S. haemolyticus* + *S. epidermidis*	1 (1.4%)	
Enterococcus faecalis + CoNS	1 (1.4%)	
Enterococcus sp. + *S. warneri* + CoNS	1 (1.4%)	
Enterococcus sp. + *S. capitis* + CoNS	1 (1.4%)	
Finegoldia magna + *Gemella morbillorum* + *Propionibacterium acnes* + *S. warneri*	1 (1.4%)	
Finegoldia magna + CoNS	1 (1.4%)	
Peptostreptococcus sp. + *S. epidermidis*	1 (1.4%)	
Anaerococcus prevotii + *S. epidermidis*	1 (1.4%)	
Micrococcus luteus + *Propionibacterium acnes* + *S. hominis* + CoNS	1 (1.4%)	
Micrococcus luteus + *S. epidermidis*	1 (1.4%)	
Micrococcus sp. + *S. epidermidis*	1 (1.4%)	
Micrococcus sp. + *S. lugdunensis*	1 (1.4%)	
Brevibacterium casei + *Aerococcus viridans*	1 (1.4%)	
Corynebacterium macginleyi + *S. epidermidis*	1 (1.4%)	
Corynebacterium mucifaciens + *S. epidermidis*	1 (1.4%)	
Corynebacterium striatum + *Peptococcus* sp. + *S. epidermidis*	1 (1.4%)	
Corynebacterium sp. + *S. epidermidis* + CoNS	1 (1.4%)	
Bacillus sp. + Group B *Streptococcus* sp.	1 (1.4%)	
Bacillus sp. + unspecified species	1 (1.4%)	
Fusobacterium sp. + *Enterococcus* sp. + *Peptostreptococcus anaerobius*	1 (1.4%)	
Proteus vulgaris + *S. epidermidis*	1 (1.4%)	
Klebsiella pneumoniae + *Corynebacterium* sp. + *Propionibacterium acnes* + unspecified anaerobe + *S. epidermidis* + CoNS	1 (1.4%)	
Ralstonia picketti + Group B *Streptococcus* sp. + *S. aureus* + *S. epidermidis*	1 (1.4%)	
S. haemolyticus + *S. epidermidis*		1 (16.7%)
Ralstonia insidiosa + *S. epidermidis*		1 (16.7%)
**Missing culture**		**1 (16.7%)**

Abbreviations: S., *Staphylococcus*; CoNS, coagulase-negative *Staphylococcus* sp. (unspecified).
Detected species are depicted once per patient.
